# Polycythemia Vera Revealed by Acute Myocardial Infarction: A Case Report

**DOI:** 10.3390/reports9010091

**Published:** 2026-03-20

**Authors:** Jéni Quintal, Raquel Flores, Tatiana Duarte, Ana Santo António, Filipe Seixo

**Affiliations:** 1 Department of Cardiology, Hospital São Bernardo, Unidade Local de Saúde da Arrábida, Rua Camilo Castelo Branco 175, 2910-549 Setúbal, Portugal; 2 Department of Internal Medicine, Hospital Egas Moniz, Unidade Local de Saúde Lisboa Ocidental, Rua da Junqueira 126, 1349-019 Lisboa, Portugal; 3 Department of Clinical Hematology, Hospital São Bernardo, Unidade Local de Saúde da Arrábida, Rua Camilo Castelo Branco 175, 2910-549 Setúbal, Portugal

**Keywords:** polycythemia vera, acute myocardial infarction, coronary thrombosis, myeloproliferative neoplasms, JAK2 V617F mutation, case report

## Abstract

**Background and Clinical Significance**: Polycythemia vera is a myeloproliferative neoplasm associated with a high thrombotic risk. Although this association is well recognized, acute coronary syndrome as the initial manifestation of polycythemia vera is rare. **Case Presentation:** We report the case of a previously healthy 57-year-old male with no conventional cardiovascular risk factors who presented with an anterior ST-elevation myocardial infarction. Coronary angiography revealed a subocclusive lesion in the left anterior descending artery, which was successfully treated with primary percutaneous coronary intervention. Initial laboratory testing showed markedly elevated hemoglobin (209 g/L) and hematocrit (64.9%), together with thrombocytosis (438 × 10^9^/L). In the absence of conventional risk factors, the combination of a single-vessel coronary lesion and marked hematologic abnormalities raised suspicion for polycythemia vera as a major contributor to coronary thrombosis. Subsequent work-up confirmed polycythemia vera based on the presence of a JAK2 V617F mutation and suppressed erythropoietin levels. The patient underwent therapeutic phlebotomy shortly after angioplasty and was subsequently started on hydroxyurea to maintain a hematocrit below 45%, together with dual antiplatelet therapy. **Conclusions:** This case highlights acute myocardial infarction as a rare initial presentation of polycythemia vera. It underscores the importance of considering polycythemia vera in patients presenting with acute coronary syndrome and unexplained erythrocytosis, while acknowledging that, in the absence of intracoronary imaging, a definitive causal link between PV and the coronary event cannot be established.

## 1. Introduction

Polycythemia vera (PV) is a chronic myeloproliferative neoplasm characterized by clonal proliferation of hematopoietic stem cells, primarily resulting in an absolute increase in red blood cell mass [1,2,3,4], often accompanied by leukocytosis and thrombocytosis [3,4]. Although morbidity is also related to progression to myelofibrosis or acute leukemia [3,4,5], the immediate clinical challenge remains the high risk of thrombotic complications. Thrombosis arises from a multifactorial prothrombotic state that is further exacerbated by the direct effect of increased cellular mass on blood rheology, with hyperviscosity impairing microvascular blood flow and increasing shear stress [1,2,3,6,7,8,9,10,11].

Although cardiovascular events are common in PV, acute coronary syndrome (ACS) as the sentinel manifestation of the disease is uncommon, albeit clinically important [8,10,11,12].

## 2. Case Description

A previously healthy 57-year-old man presented to the emergency department with constrictive chest pain that began after a 10 km walk and progressively worsened over three days. He had no conventional cardiovascular risk factors and no family history of coronary artery disease or sudden cardiac death.

**Figure 1:**
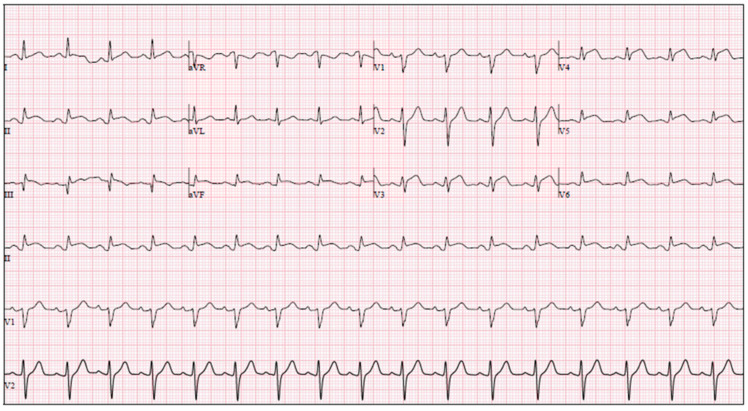
Electrocardiogram at admission showing sinus rhythm, 75 bpm, predominant ST-segment elevation in leads V3–V6, and mild J-point elevation in leads II, III, and aVF.

On arrival, he was hypertensive (blood pressure 169/120 mmHg), with a normal heart rate and peripheral oxygen saturation of 91–95% on room air. Physical examination was otherwise unremarkable. The initial electrocardiogram (ECG) showed sinus rhythm with predominant ST-segment elevation in leads V3–V6 and mild J-point elevation in leads II, III, and aVF, consistent with an extensive anterior STEMI (Figure 1).

He received loading doses of aspirin and unfractionated heparin before expedited transfer for coronary angiography. Procedural timings were as follows: pain-to-door time, 40 min; door-to-balloon time, 5 min.

**Figure 2:**
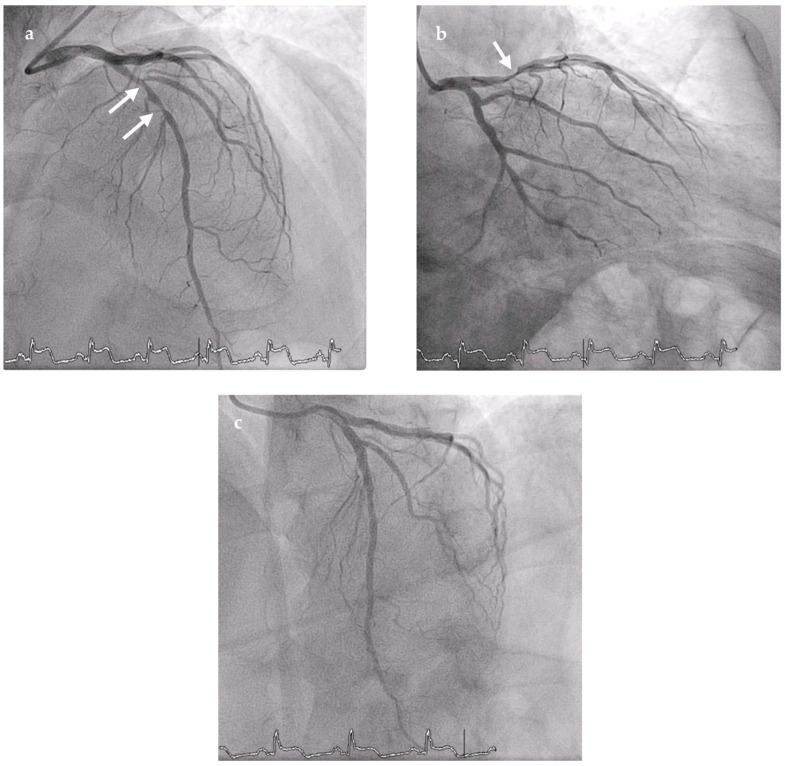
Coronary angiography: (**a**,**b**) pre-intervention views demonstrating a sub-occlusive culprit lesion in the proximal left anterior descending (LAD) artery (arrows) with ostial involvement of the first diagonal branch (TIMI 2); (**c**) final angiogram after primary percutaneous coronary intervention, showing resolution of the culprit stenosis and restored anterograde flow in the LAD (TIMI 3).

Angiography revealed a subocclusive proximal left anterior descending (LAD) artery lesion with reduced antegrade flow (TIMI 2), and a primary percutaneous coronary intervention (PCI) with thrombus aspiration and angioplasty restored flow (TIMI 3; Figure 2). Transthoracic echocardiography performed shortly thereafter demonstrated apical akinesia with preserved left ventricular ejection fraction, normal right ventricular function (TAPSE 18 mm), an estimated systolic pulmonary artery pressure of 28 mmHg, and no significant valvular disease.

Initial laboratory testing showed marked erythrocytosis and thrombocytosis, with hemoglobin of 209 g/L, hematocrit of 64.9%, and platelet count of 438 × 10^9^/L (Table 1). The lipid profile showed triglycerides of 0.97 mmol/L, HDL-cholesterol of 1.06 mmol/L, and LDL-cholesterol of 3.47 mmol/L. High-sensitivity troponin I peaked at 17,837.8 pg/mL (upper limit of normal, 28.9–39.2 pg/mL). In a patient presenting with an acute arterial thrombotic event in the absence of conventional cardiovascular risk factors, the single-vessel culprit pattern together with the hematologic abnormalities prompted evaluation for an underlying myeloproliferative neoplasm, particularly PV.

Despite initial hydration, erythrocytosis and the elevated hematocrit persisted, prompting therapeutic phlebotomy 8 h after angiography. Subsequent etiologic evaluation identified a JAK2 V617F mutation and a markedly suppressed serum erythropoietin level (<0.6 mU/mL; reference interval 2.6–19 mU/mL), fulfilling the 2016 World Health Organization (WHO) diagnostic criteria for PV. To further exclude secondary or paraneoplastic causes of erythrocytosis, such as chronic hypoxia, smoking, or obstructive sleep apnea, a comprehensive work-up was performed, including chest X-ray, chest CT, and abdominal ultrasound, which showed no evidence of malignancy, renal masses, or other occult pathology.

Throughout hospitalization, the patient remained hemodynamically stable (Killip class I) and reported no aquagenic pruritus, headache, visual disturbances, or symptoms suggestive of additional arterial or venous thrombosis. No further hypertensive episodes were recorded. The hospital course was uneventful, and he was discharged after 72 h. During a 1-year follow-up, he remained asymptomatic, with no recurrent cardiovascular events.

## 3. Discussion

Acute myocardial infarction as the sentinel manifestation of PV is uncommon, with fewer than 15 cases reported in the literature [6,10,11,12,13]. This case illustrates the importance of carefully integrating clinical, angiographic, and hematologic data to uncover an otherwise occult myeloproliferative neoplasm.

PV carries a substantial symptom burden, including dizziness, headache, visual disturbances, erythromelalgia, distal paresthesia, and acrocyanosis, all of which may significantly impair quality of life [2,3,4]. It is also associated with considerable morbidity and mortality, with hemorrhagic complication rates of approximately 4% [2,6] and thrombotic complication rates ranging from 20% to 40% during follow-up, about half of which are arterial events [1,4,6]. Although long-term series suggest that myocardial infarction occurs in 11.4% of patients with PV over 10 years [10], STEMI as the presenting event remains exceptionally rare.

In the present case, the combination of single-vessel STEMI, marked erythrocytosis, thrombocytosis, and JAK2 V617F positivity strongly suggests that PV was an important contributor to coronary thrombosis. However, PV cannot be established as the sole causal mechanism with certainty. Although the LDL-cholesterol elevation was only mild and angiography did not show diffuse obstructive disease, the possibility of an underlying atherosclerotic substrate cannot be fully excluded.

Several mechanisms may plausibly have converged to precipitate coronary thrombosis in this setting. Elevated hematocrit increases whole-blood viscosity, reduces microvascular perfusion, and amplifies shear stress, thereby favoring platelet activation and thrombin generation [5]. In addition, PV is associated with qualitative platelet and leukocyte abnormalities that promote adhesion and intravascular thrombosis, even when angiographically visible atherosclerosis is limited. JAK2-driven signaling may further enhance endothelial activation and procoagulant pathways [7]. Red-cell adhesiveness, mediated in part by Lu/BCAM activation downstream of JAK2 V617F, provides an additional prothrombotic substrate [7]. Neutrophil extracellular traps (NETs) have also been implicated in myeloproliferative neoplasms and may contribute to both arterial and venous events [8].

The mechanisms make PV a biologically plausible contributor to the acute LAD event observed in our patient. However, because intracoronary imaging (OCT or IVUS) was not performed, plaque rupture or plaque erosion cannot be definitively excluded at the culprit lesion. Likewise, because the non-culprit vessels were assessed only angiographically, subclinical atherosclerosis in other territories cannot be ruled out beyond doubt. Accordingly, PV should be interpreted as a highly plausible contributor to thrombosis in this case, rather than as a definitively proven sole cause.

Although the mutational landscape of myeloproliferative neoplasms also includes other alterations, such as CALR (calreticulin) or EPOR (erythropoietin receptor) variants, these were not investigated in this case. This was not considered a major limitation because CALR mutations are predominantly associated with essential thrombocythemia and primary myelofibrosis and are generally mutually exclusive with JAK2, whereas EPOR mutations are usually investigated in primary familial erythrocytosis when JAK2 is negative, and erythropoietin levels are subnormal. In our patient, the presence of the JAK2 V617F mutation, together with suppressed serum erythropoietin (<0.6 mU/mL), was sufficient to confirm the diagnosis of PV according to the 2016 WHO criteria. The borderline oxygen saturation at admission (91–95%) was attributed to the acute STEMI and initial hemodynamic stress rather than chronic hypoxia, as values normalized during hospitalization without oxygen therapy. Abdominal ultrasound showed no evidence of renal tumors or significant splenomegaly. According to the 2016 WHO criteria, a bone marrow biopsy may be omitted in cases of sustained absolute erythrocytosis (hemoglobin >18.5 g/dL in men) when both a JAK2 mutation and subnormal erythropoietin levels are present, as in our patient.

The diagnosis of PV was established according to the 2016 WHO criteria (Table 2), based on absolute erythrocytosis, JAK2 V617F positivity, and subnormal erythropoietin levels. Bone marrow biopsy was not required because sustained absolute erythrocytosis, together with a clonal marker and low erythropoietin, is sufficient for diagnosis [14]. In addition, the biochemical profile and the subsequent oxygen-saturation trajectory argued against secondary causes of erythrocytosis; a low erythropoietin level is particularly useful in distinguishing PV from hypoxia-driven or exogenous etiologies, including smoking, obstructive sleep apnea, obesity-hypoventilation syndrome, and chronic hypoxia [2].

Following prompt reperfusion of the proximal LAD, management focused on reducing PV-related thrombotic risk. Persistent erythrocytosis despite hydration prompted therapeutic phlebotomy, targeting a hematocrit below 45%, in keeping with CYTO-PV5. Low-dose aspirin is recommended in PV and may be continued alongside the post-PCI antiplatelet regimen when not contraindicated [3,4,5]. For long-term prevention, contemporary frameworks classify patients as high risk if they are aged ≥ 60 years or have a history of thrombosis. Although our patient was younger than 60 years, the index myocardial infarction fulfilled the latter criterion, thereby supporting initiation of cytoreductive therapy to reduce recurrent events and the need for phlebotomy [3,4,5]. In line with current risk-stratification models, including the 2024 update by Tefferi and Barbui, our patient was categorized as high-risk because of a major arterial thrombotic event, regardless of age. This classification justified prompt initiation of cytoreductive therapy alongside standard post-PCI care. Hydroxyurea is the preferred first-line agent, with pegylated interferon-α as an alternative, particularly in younger patients or when fertility and cytopenia considerations are relevant [2,4]. Accordingly, dual antiplatelet therapy was maintained, and hydroxyurea was initiated to maintain hematocrit below 45%, with coordinated follow-up in cardiology and hematology.

Regarding prognosis, risk factors associated with reduced survival include advanced age, leukocytosis, and prior thrombosis [2,4]. In addition, prior arterial events, hyperlipidemia, and hypertension have been identified as predictors of subsequent arterial thrombosis in multivariable analysis by Cerquozzi et al. [9].

This case emphasizes that, in patients with myocardial infarction and unexplained erythrocytosis, especially when the angiographic pattern is limited to a single culprit vessel, screening for PV with complete blood count trends, serum erythropoietin, and JAK2 testing is simple and may help prevent recurrent arterial or venous events through timely cytoreduction and hematocrit control.

As a single case report, this work is inherently limited by the inability to generalize its findings. An additional and important limitation is that the causal role of PV in the acute coronary event cannot be established with certainty. Intracoronary imaging was not evaluated beyond angiography. Therefore, underlying plaque disruption or subclinical atherosclerosis cannot be excluded beyond doubt, and a bystander role of PV remains possible. For this reason, we interpret PV as a highly plausible contributory mechanism rather than a definitively proven sole cause of thrombosis in this case.

## 4. Conclusions

In conclusion, PV should be suspected in patients presenting with acute coronary syndrome and unexplained erythrocytosis, particularly in the absence of conventional cardiovascular risk factors. Early recognition through routine blood count analysis and timely hematologic treatment may help reduce the risk of recurrent thrombotic events. Nevertheless, in the absence of intracoronary imaging, the causal contribution of PV to the index coronary event should be interpreted with appropriate caution.

## Figures and Tables

**Table 1:** The patient’s clinical laboratory data.

Blood Test	Admission	Discharge	1st Cardiology Appointment	1 Year Follow-Up
Hemoglobin (g/L)	209	179		151
Haematocrit (%)	64.9	55.2		45.0
Erythrocytes (×10^12^/L)	7.91	6.77		4.2
Leucocytes (10^9^/L)	9.0	6.5		4.6
Platelets (10^9^/L)	438	448		355
LDL cholesterol (mmol/L)	3.47			
HDL cholesterol (mmol/L)	1.06			
Triglycerides (mmol/L)	0.97			
hs-Troponin I (pg/mL)	17,837.8 (peak)	2752		
Erythropoietin (mU/mL)		<0.6		
JAK2 kinase mutation			JAK-2 V617F mutation: positive	

hs—high sensitivity.

**Table 2:** 2016 World Health Organization diagnostic criteria for polycythemia vera ^1^.

**Major Criteria**
Hemoglobin > 16.5 g/dL (men)/>16.0 g/dL (women), or hematocrit > 49% (men)/>48% (women), or increased red cell mass (RCM)
Bone marrow biopsy showing age-adjusted hypercellularity with trilineage growth (panmyelosis), including prominent erythroid, granulocytic, and megakaryocytic proliferation with pleomorphic, mature megakaryocytes
Presence of JAK2 or JAK2 exon 12 mutation
**Minor criteria**
Subnormal serum erythropoietin level
**A diagnosis of PV can be established by meeting all 3 major criteria or the first two major criteria plus the one minor criterion**

^1^ Table adapted from Barbui et al. *Blood Cancer J* 2018; 8:15 [14].

## Data Availability

The original data presented in the study are included in the article, further inquiries can be directed to the corresponding authors.

## Abbreviations

The following abbreviations are used in this manuscript:

ACS	Acute Coronary Syndrome
LAD	Left Anterior Descending Artery
PCI	Percutaneous Coronary Intervention
PV	Polycythemia vera
STEMI	ST-Elevation Myocardial Infarction
